# The Effect of High-Intensity Interval Exercise on Short-Term Glycaemic Control, Serum Level of Key Mediator in Hypoxia and Pro-Inflammatory Cytokines in Patients with Type 1 Diabetes—An Exploratory Case Study

**DOI:** 10.3390/nu15173749

**Published:** 2023-08-27

**Authors:** Barbara Hall, Aleksandra Żebrowska, Marcin Sikora, Szymon Siatkowski, Anna Robins

**Affiliations:** 1 School of Physiological and Medical Sciences, Department of Physiology, The Jerzy Kukuczka Academy of Physical Education, Mikolowska Street 72a, 40-065 Katowice, Poland; a.zebrowska@awf.katowice.pl (A.Ż.); m.sikora@awf.katowice.pl (M.S.); 2 Institute of Healthy Living, The Jerzy Kukuczka Academy of Physical Education, Mikolowska Street 72a, 40-065 Katowice, Poland; s.siatkowski@awf.katowice.pl; 3 School of Health and Society, University of Salford, Allerton Building, 43 Crescent, Salford M5 4WT, UK; a.robins@salford.ac.uk

**Keywords:** type 1 diabetes, high-intensity interval exercise, glycaemic control, hypoxia, inflammation

## Abstract

Type 1 diabetes (T1D) is associated with hyperglycaemia-induced hypoxia and inflammation. This study assessed the effects of a single bout of high-intensity interval exercise (HIIE) on glycaemia (BG) and serum level of pro-inflammatory cytokines, and an essential mediator of adaptive response to hypoxia in T1D patients. The macronutrient intake was also evaluated. Nine patients suffering from T1D for about 12 years and nine healthy individuals (CG) were enrolled and completed one session of HIIE at the intensity of 120% lactate threshold with a duration of 4 × 5 min intermittent with 5 min rests after each bout of exercise. Capillary and venous blood were withdrawn at rest, immediately after and at 24 h post-HIIE for analysis of BG, hypoxia-inducible factor alpha (HIF-1α), tumour necrosis factor alpha (TNF-α) and vascular-endothelial growth factor (VEGF). Pre-exercise BG was significantly higher in the T1D patients compared to the CG (*p* = 0.043). HIIE led to a significant decline in T1D patients’ BG (*p* = 0.027) and a tendency for a lower BG at 24 h post-HIIE vs. pre-HIIE. HIF-1α was significantly elevated in the T1D patients compared to CG and there was a trend for HIF-1α to decline, and for VEGF and TNF-α to increase in response to HIIE in the T1D group. Both groups consumed more and less than the recommended amounts of protein and fat, respectively. In the T1D group, a tendency for a higher digestible carbohydrate intake and more frequent hyperglycaemic episodes on the day after HIIE were observed. HIIE was effective in reducing T1D patients’ glycaemia and improving short-term glycaemic control. HIIE has the potential to improve adaptive response to hypoxia by elevating the serum level of VEGF. Patients’ diet and level of physical activity should be screened on a regular basis, and they should be educated on the glycaemic effects of digestible carbohydrates.

## 1. Introduction

Chronically elevated glycaemia in diabetes mellitus (DM) has been shown to induce a pro-inflammatory phenotype of endothelium and immune cells of primarily myeloid origin [1]. In a hyperglycaemic environment (fasting glycaemia > 125 mg/dL and 2 h postprandial glycaemia > 180 mg/dL), macrophages, which are the main source of inflammatory cytokines, respond by enhancing secretion of mainly pro-inflammatory cytokines such as tumour necrosis factor alpha (TNF-α) [1,2]. TNF-α is a key pro-inflammatory cytokine, which is involved in systemic inflammation, but it also has an impact on physiological metabolic homeostasis such as glucose metabolism [3]. An abnormally elevated and sustained secretion of TNF-α is associated with autoimmune and other inflammatory diseases [4,5]. When compared to healthy individuals, increased levels of TNF-α have been found in individuals with newly diagnosed Type 1 d0iabetes (T1D), and the cytokine response has been shown to correlate positively with disease duration and the patient’s age [6]. TNF-α is also secreted by the endothelial cells in response to their persistent exposure to hyperglycaemia, which eventually leads to endothelial dysfunction (ED). ED is associated with reduced nitric oxide (NO) bioavailability and, consequently, reduced vasodilation, oxidative stress, increased permeability of the endothelial barrier and dysregulated expression of growth factors and pro-inflammatory cytokines, including TNF-α, vascular endothelial growth factor (VEGF) and the transcription factor hypoxia-inducible factor alpha (HIF-1α) [7,8,9]. ED initiates the development of micro- and macrovascular complications in T1D patients [10]. 

VEGF is an established stimulator of physiological and pathophysiological angiogenesis [11,12], whilst it also acts as a pro-inflammatory cytokine by increasing the permeability of the endothelial barrier and by being chemotactic for monocytes [13,14]. The expression and activation of HIF-1α as a transcription factor is regulated by hypoxia and glucose [15,16]. Hypoxia, which is defined as “insufficient cellular level of oxygen”, develops in most tissues of patients with T1D [17]. It has been proposed that hyperglycaemia induces hypoxia in endothelial cells by elevating the rate of glycolysis, thus increasing the generation of mitochondrial ROS, which in turn suppress the expression of aquaporin-1 (AQP-1) [18], a water channel that also facilitates oxygen diffusion across the cellular membrane [19]. The reduced expression of AQP-1 will lead to hypoxia and increased secretion of HIF-1α. Additionally, hypoxia in T1D can develop due to chronic exposure of blood cells, such as neutrophils and macrophages, to hyperglycaemia, which causes glycation of their protein molecules. This would adversely affect their structure and diapedesis ability, which in turn would cause the cells to plug the vessel lumen, causing hypoxia of the endothelial cells [20]. 

Hyperglycaemia-induced chronic inflammation, sustained by the elevated levels of pro-inflammatory cytokines, leads to the development of life-threating micro- and macrovascular complications in both main types of DM [21,22]. Therefore, sufficient glycaemic control is of paramount importance in the management of T1D [23]. T1D treatment should not only consist of insulin therapy, but also adequate nutrition and regular physical exercise [24,25,26,27]. 

It has been shown that knowledge and understanding of T1D dietary management is lacking [28], and not all T1D patients are aware that digestible (glycaemic) carbohydrates are fundamental macronutrients for influencing BG and insulin concentrations [29]. Foods such as potatoes, refined grains and those containing added sugars are harmful and have been linked to higher glycaemia, inflammation and cardiometabolic risk [30,31]. Furthermore, it has been shown that less than a fifth of adults with T1D manage to meet physical activity recommendations [32,33], despite evidence that regular physical activity in this population group provides many physiological and psychological benefits [34], whilst reducing daily insulin requirements [35]. 

When compared with moderate-intensity continuous training (MICT), the popularity of high-intensity interval single exercise (HIIE) and training (HIIT) has increased due to its time-saving characteristics, as well as the same, or even greater, effectiveness in improving cardiovascular risks factors in sedentary people, patients with coronary artery disease, heart failure and those with high cardiovascular risk [36,37,38,39,40]. HIIE has been shown to be effective in reducing glycaemia in T1D and T2D patients to a lower [41] or a greater [42] extent than MICT. However, it has also been demonstrated that HIIE can lead to an increase in glycaemia during or immediately after its completion in T1D patients [43]. This can be attributed to the higher release of counter-regulatory hormones, such as catecholamines, glucagon and cortisol [44]. Additionally, after the initial elevation in BG, there is a risk of late-onset (up to 24–48 h post-exercise) hypoglycaemia, and the fear of this often stops patients with T1D from exercising [41,45].

In this study, we aimed to assess the acute and up to 24 h post-exercise effects of a single bout of HIIE on glycaemia, serum level of pro-inflammatory cytokines and a transcriptional factor that mediates response to hypoxia in T1D patients. In addition, we also evaluated the participants’ macronutrient intake and compared it against dietary recommendations for this population group. 

## 2. Materials and Methods

### 2.1. Participants

**Figure 1:**
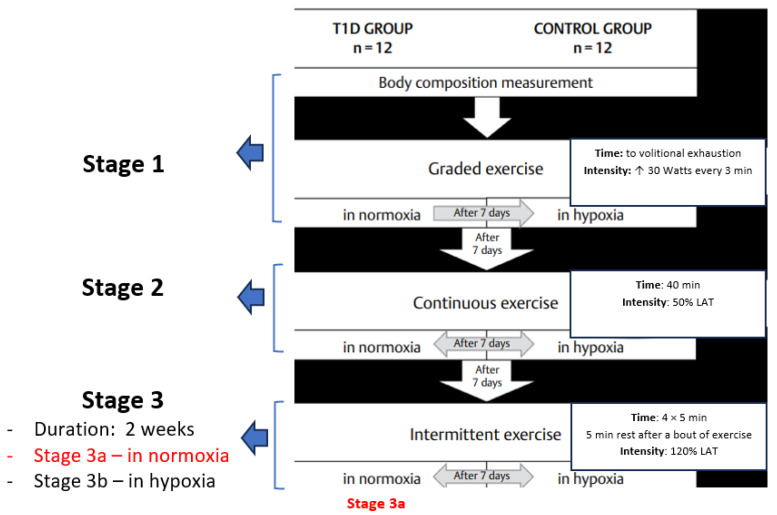
The research project’s exercise protocol. Stage 1—participants performed a graded exercise test; Stage 2—participants performed a moderate-intensity continuous exercise test; Stage 3—participants performed a high-intensity interval exercise test (HIIE). All exercise tests were performed in normoxia and normobaric hypoxia (FiO_2_ = 15.1%). The present paper describes the results of the intermittent exercise test (HIIE) in normoxia only (Stage 3a).

Participants suffering from T1D for about 12 years (12.1 ± 6.0) were recruited at the Diabetes Clinic of the Silesian Centre in Poland and took part in the research project, which consisted of three stages, the first two of which have already been published [46,47]. The current paper presents the findings of the third stage of the project, which was completed by nine participants with T1D (T1D group) (Figure 1). 

All T1D participants adhered to intensive insulin (INS) therapy; half of them were using continuous subcutaneous INS infusion (rapid-acting INS: Humalog, NovoRapid or Apidra) and the other half multiple daily INS injections with long-acting (Lantus or Levemir) and rapid-acting (NovoRapid) INS. The maximum glucose-lowering effect of all three rapid-acting types of INS occurs between 1 and 3 h, and the INS effect lasts 3–5 h. For long-acting INS, the peak effect is observed after 6–14 h (Levemir only, with Lantus having no peak effect), and the duration of action is between 16 and 20 (Levemir) and 20 and 24 h (Lantus) [48]. Only participants free of diabetic complications, with no history of other (not-diabetes related) metabolic diseases were enrolled. The other inclusion criteria were as follows: no participation in another trial, having not had the common cold, influenza, or other infections up to 1 week prior to the study, being a non-smoker and having good exercise tolerance confirmed by the direct measurement of peak oxygen uptake (VO_2_peak) [46,47] and glycated haemoglobin (HbA1c) < 8.0% (Table 1). The medical history and information regarding diabetes aetiology of the study participants were prepared by medical personnel of the Silesian Centre in Poland. The control group consisted of nine healthy individuals, without impaired glucose tolerance, and of a similar body composition (Table 1). 

Before the study, the procedure and the related risks were explained to all potential participants prior to taking consent. They were also informed that the study was voluntary, and they could withdraw from it at any time without giving reasons. Written informed consent was obtained from all participants, and they were asked to abstain from exercise and the consumption of alcohol and caffeinated drinks 24 h prior to the tests. Their diet, fasting glycaemia and insulin dosage were monitored for the entire duration of the study. Composition of the diet was calculated with dedicated software (Dietus, B.U.I. InFit, Warszawa, Poland). 

### 2.2. Study Protocol

All individuals were asked to arrive at the laboratory at least 2 h after their breakfast. The T1D patients were asked to have their pre-exercise glycaemia within the range of 100–160 mg/dL and were advised to reduce their basal insulin infusion by 50% before the exercise tests. The participant’s body mass and composition were determined by means of Bioelectrical Impedance Analysis (BIA) (InBody 220 Data Management System, Biospace, Seoul, Republic of Korea).

The participants completed one high-intensity (120% lactate threshold: LAT, which was determined with the Dmax method [49] and corresponded to ≈90 ± 5% of maximum heart rate (HRmax)) interval exercise session (HIIE): 4 × 5 min intermittent bouts of cycling with 5 min rest after each bout of exercise) at the laboratory with the ambient conditions of 21 °C and 60% relative humidity. 

### 2.3. Biochemical Analyses

Venous and capillary blood samples were taken from the cubital vein and a fingertip, respectively, at rest (pre-HIIE), immediately after the cessation of HIIE (post-HIIE) and at 24 h post-exercise. 

Blood glucose concentration was measured in the capillary blood with the enzymatic method (glucose dehydrogenase) (Glucose 201⁺, HemoCue, Ängelholm, Sweden). 

To measure the levels of selected pro-inflammatory cytokines, TNF-α and VEGF, as well as transcription factor HIF-1α, venous blood samples were left to clot at room temperature for 30 min and centrifuged for 15 min at 1000× *g*. The obtained serum was kept frozen at 80 °C (for a period no longer than 8 months) without repeated freezing. Serum TNF-α was assessed using Immuno Assays, DIAsource, Louvain-la-Neuve, Belgium. The intra- and inter-assay coefficients of variation (CV) were 6.3 and 3.3%, respectively. Serum levels of HIF-1α and VEGF were measured with an enzyme-linked immunosorbent assay ELISA kit (BlueGene Biotech Co., Ltd., Shanghai, China). The intra- and inter-assay coefficients of variations for HIF-1α and VEGF were <4.4% and <5.6%, respectively. 

The T1DM patients were asked to record their BG, insulin dosages, and hypo- or hyperglycaemia events up to 24 h after the HIIE (patients’ diary). A BG value of <70 mg/dL indicated hypoglycaemia, and a fasting BG value of >125 mg/dL or 2 h postprandial BG of >180 mg/dL was diagnosed as hyperglycaemia [50]. The percentage change in BG (% change) was calculated using the following equation:

% change = ((V_2_ − V_1_))/(V_1_) × 100

where:

V_1_ represents the pre-HIIE value;
V_2_ represents the post-HIIE value.

### 2.4. Statistical Methods

The statistical package (StatSoft Poland, 12.0) was used for data processing and analysis. The Shapiro–Wilk, Levene’s and Mauchly’s tests were used to verify data normality, homogeneity and sphericity, respectively. Normally distributed data are presented as means and standard deviations. A one-way analysis of variance was used to compare the somatic variables between the studied groups and a two-way repeated measures ANOVA to verify the differences between the groups (T1D vs. control group) and HIIE (pre-HIIE vs. post-HIIE). The effect size partial eta squared (η2) for the differences between the groups and between pre- and post-HIIE was also calculated. The criteria to interpret the magnitude of the effect sizes (ES) were <0.2 trivial, 0.2–0.6 small, 0.6–1.2 moderate, 1.2–2.0 large and >2.0 very large. The significance of the differences between the studied variables was verified with the Bonferroni post hoc test, and *p* < 0.05 indicated statistical significance.

### 2.5. Ethics

The study protocol received approval from the Ethics Committee of the Jerzy Kukuczka Academy of Physical Education in Katowice, Poland (the committee resolution number 3/2011; date of approval 26 October 2011) and conformed to the standards set by the Declaration of Helsinki.

## 3. Results

### 3.1. Participants’ Somatic and Physiological Characteristics 

There were no significant differences in body mass index (BMI), percentage of body fat (%BF) and fat-free mass (FFM) between the T1D and control groups (Table 1). The mean %BF was low in both groups [51]. In T1D, one male participant was found with %BF (24.0%) above that recommended for the age range (>22%), and his BMI (29.9 kg/m^2^) indicated he was overweight (≥25.0 kg/m^2^). There was a non-significant tendency for age difference (*p* = 0.07) between the studied groups. The glycated haemoglobin (HbA1C), which reflects an average glycemia over approximately 3 months preceding the measurement, was above the recommended level (HbA1C < 7.0%) in seven out of nine patients (78%) [23].

The assessment of aerobic fitness was based on VO_2_peak and considered age and gender [52]. The determination of the VO_2_peak was assessed during stage 1 of the project [46]. In the T1D group, 33.3% (*n* = 3) of participants were found with a VO_2_ indicating poor, 44.4% excellent (*n* = 3) and 11% (*n* = 1) superior aerobic fitness, whilst the remaining participants had a good level of aerobic fitness. In the control group, one participant had poor, one had good, whilst 77.8% (*n* = 7) had excellent and superior levels of aerobic fitness.

### 3.2. Macronutrients Intake

The one-way ANOVA did not reveal any significant differences in macronutrient intake between the studied groups, although there was a tendency for lower carbohydrate and energy consumption in the T1D group compared to the control group. Both groups consumed higher (>20% energy intake) and lower (<20% energy intake) than recommended [53] amounts of protein and fat, respectively.

### 3.3. Glycaemia in Response to HIIE

**Figure 2:**
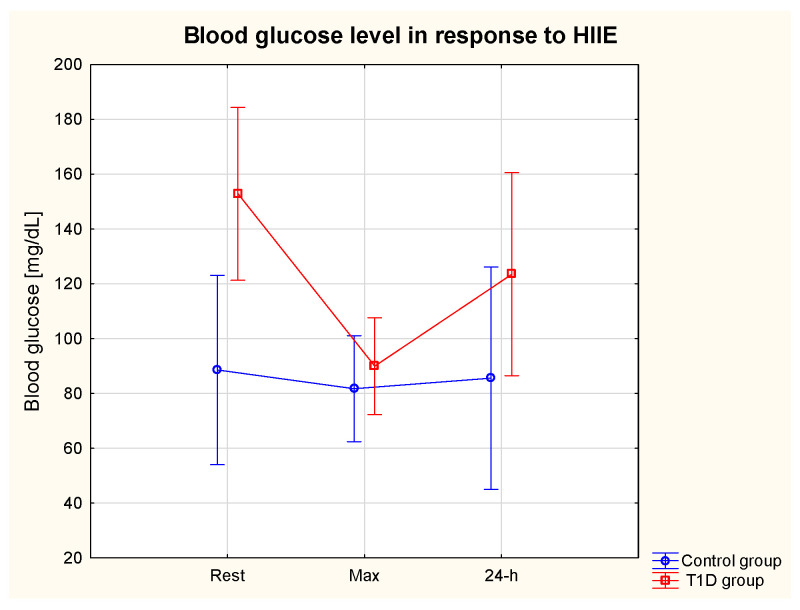
Serum glucose level at rest, immediately after and at 24 h post-exercise period in the T1D and control groups.

There was a significant effect of group (T1D vs. control group) on glycaemia (η2 = 0.50, *p* = 0.01) (ES = small). The post hoc analysis revealed a significantly higher resting BG (BG rest) in the T1D compared to the control group (*p* = 0.04) and a significant decline in T1D patients’ BG in response to HIIE (pre-HIIE: 152.8 ± 92.0 vs. immediately post-HIIE: 90.0 ± 55.7 mg/dL) (*p* = 0.03), and the BG % change was 41.11%. There was a tendency (*p* = 0.59) towards lower BG at 24 h post-HIIT in comparison with pre-HIIE (123.5 ± 52.0 vs. 152.83 ± 92.0 mg/dL, respectively) in T1D. In the control group, HIIE led to a slight decrease in the BG (%change 7.7%) (Figure 2).

### 3.4. Glycaemic Control in 24 h Post-Exercise Period 

Based on the patients’ diary, fasting glycaemia, number of glycaemic disorders, insulin administration and intake of digestible carbohydrates (CHO; with exclusion of dietary fibre) were compared on the day of (day 1) and the day after (day 2) the HIIE (Table 2). ANOVA did not reveal a significant effect of day on the above-mentioned variables. There was a tendency for a lower fasting glycaemia on day 2 compared to day 1, and a higher digestible CHO intake on day 2 in comparison with day 1 (Table 2). Hypoglycaemia developed at least once in 20% T1D patients on day 1 with the same % on day 2, whilst hyperglycaemic disorders were more frequent and developed (between 1 and 4 times) in 60% of T1D patients on day 1 and 80% T1D on day 2.

### 3.5. HIF-1α, TNF-α and VEGF in Response to HIIE

Table 3 presents the serum levels of the transcription factor HIF-1α the pro-inflammatory cytokines (TNF-α and VEGF) in response to HIIE.

#### 3.5.1. HIF-1α

**Figure 3:**
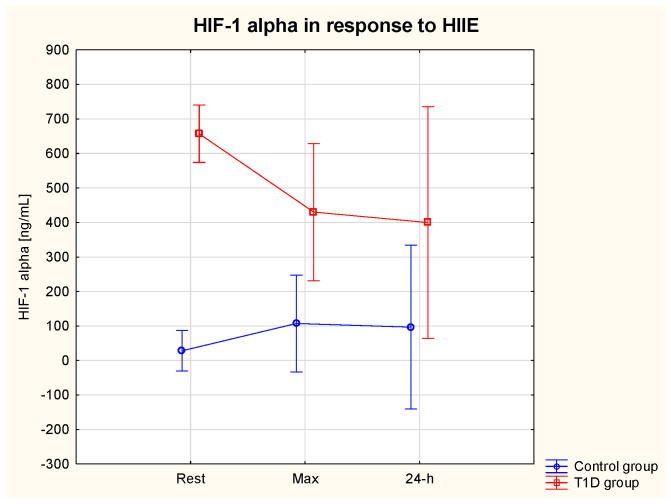
HIF−1 alpha (α) serum level at rest, immediately after and at 24 h post-exercise period in the T1D and control group.

The ANOVA revealed a significant effect of the group on HIF-1α (η2 = 0.86, *p* = 0.01) (ES = moderate). Serum HIF-1α rest was significantly higher in the T1D compared to the control group (657.0 ± 210.4 vs. 28.3 ± 11.5 ng/mL) (*p* = 0.01) (Table 2). There was a significant interaction effect (Group * HIIE) on HIF-1α (η2 = 0.65, *p* = 0.01) (ES = moderate) and a tendency for lower HIF-1α max (*p* = 0.13) and HIF-1α 24 h (*p* = 0.07) in comparison with HIF-1α rest in the T1D group (Figure 3).

#### 3.5.2. TNF-α

**Figure 4:**
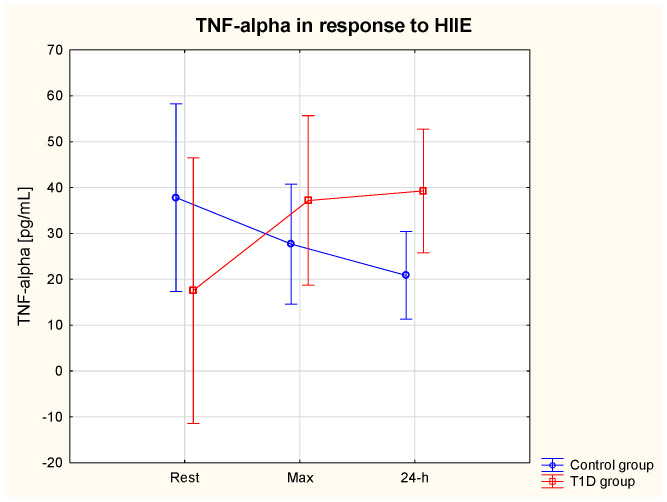
TNF−alpha (α) serum level at rest, immediately after and at 24 h of post-exercise period in the T1D and control group.

ANOVA showed a significant interaction effect (group * HIIE) on serum TNF-α (η2 = 0.56, *p* = 0.04) (ES = small). The post hoc analysis did not reveal significant differences between the group or in response to HIIE. Only a tendency for a TNF-α increase in response to HIIE was seen in the T1D group, whereas TNF-α tended to decrease in the post-exercise period in healthy participants (Table 3). The resting levels of TNF-α tended to be slightly higher in the control vs. T1D group (Figure 4).

#### 3.5.3. VEGF

**Figure 5:**
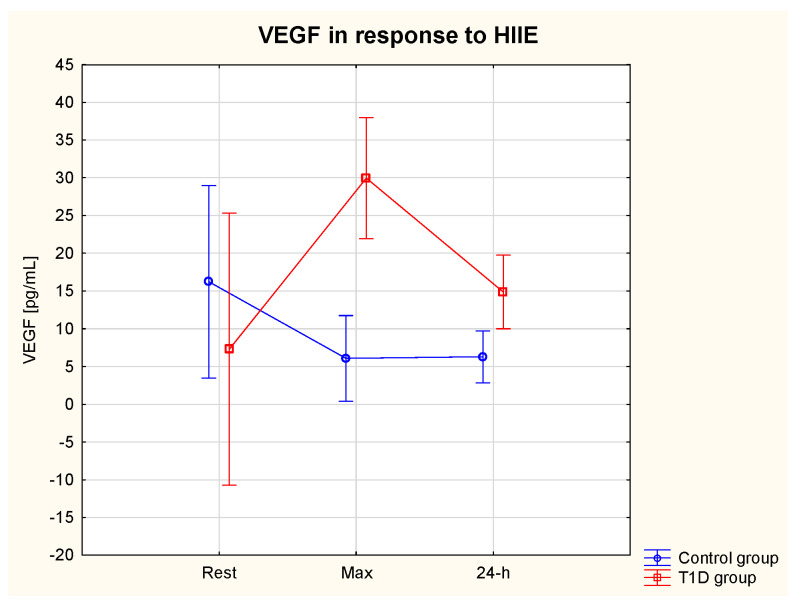
VEGF serum level at rest, immediately after and at 24 h of post-exercise period in the T1D and control group.

The ANOVA revealed a significant effect of the group (η2 = 0.75, *p* = 0.02) (ES = moderate) and an interaction effect (group * HIIE) η2 = 0.67, *p* = 0.01) (ES = moderate) on serum VEGF. VEGF max was significantly lower in the T1D compared to the control group (*p* = 0.02) (Table 3). There was also a tendency (*p* > 0.05) for lower resting levels of VEGF in T1D vs. control group (Figure 5).

## 4. Discussion

The acute and up to 24 h effects of HIIE on glycaemia and the level of a transcriptional factor and selected pro-inflammatory cytokines were measured in patients with T1D with moderate glycaemic control [23] and healthy individuals. The major finding of this study is that HIIE significantly reduced patients’ BG to a safe level and there was a tendency for lower BG in the 24 h post-exercise compared to the baseline BG. HIIE did not lead to significant changes, although there was a tendency for lower HIF-1α and increased VEGF immediately after HIIE and at 24 h post-exercise. There was also a tendency for TNF-α to increase in response to HIIE in the T1D group only. 

Glycaemia control in T1D is a constant challenge and patients with T1D are at increased risk of acute and chronic diabetes-related complications. In addition to insulin therapy, an appropriate diet and regular exercise can help patients manage their glycaemia more efficiently [24,32]. However, some individuals with T1D choose not to exercise due to the increased probability of experiencing a hypoglycaemic event [41,54], or for other reasons, which can be seen as typical barriers to exercise, such as laziness [55], stressful work conditions or lack of discipline [56], or a misunderstanding in terms of its effectiveness [57]. According to the current physical activity and exercise guidelines, most adults with T1D should engage in 150 min or more of moderate to vigorous intensity aerobic activity and/or exercise per week, and that shorter durations (minimum 75 min/week) of vigorous aerobic or anaerobic interval exercise may be sufficient for younger and more physically fit individuals [23]. Our study shows that patients with lower levels of fitness (33.3% of participants were found with a VO_2_peak indicating poor aerobic fitness) were capable of performing the HIIE that was “near maximal” effort (i.e., 90–95% of HR max/120% of LAT). However, the question of whether these patients would continue exercising at a high intensity and would choose HIIE over MICT remains. It needs to be added that prescription of HIIE needs to be individualised, and potential diabetes-related vascular complications such as neuropathy, which increases the risk of foot ulcers during, e.g., running, and other factors, including T1D duration, must be considered [58].

The intensity of any exercise undertaken is an important consideration in T1D, and there is evidence to show that HIIE reduces the risk of hypoglycaemia when compared to moderate continuous exercise [59,60]. This can be explained by a greater reliance on intramuscular glycogen and phosphagens over blood glucose as an energy source [61], as well as augmented gluconeogenesis [62]. 

Our study findings confirm the effectiveness of HIIE in reducing glycaemia in T1D patients, as a significant BG reduction was observed immediately after HIIE. In addition, the beneficial effect of HIIE seemed to be maintained until the next day, as there was a tendency for lower fasting BG in the T1D group. The acute and up-to-24-h BG-lowering effect of the HIIE could be attributed to an increased translocation of the glucose transporter type 4 (GLUT-4) from the sarcoplasm to sarcolemma, which facilitates the transport of glucose into the muscle cell, and also by augmented blood perfusion, thus increasing a rate of glucose dispersion into the muscle interstitial space [63]. Moreover, muscle glycogen stores diminish during HIIE, leading to a glucose/glucose 6-phosphate gradient that favours additional glucose entry into the skeletal muscle, and this and other molecular changes induced by exercise can be maintained for up to 48 h [64]. Despite these BG-lowering effects of HIIE, T1D patients experienced more episodes of hyperglycaemia on the day following, rather than on the day of HIIE (day 2 vs. day 1), which could be associated with a tendency for a higher glycaemic CHO intake on day 2 (190.5 ± 57.7 g/day) compared to day 1 (157.0 ± 104.2 g/day). In another study, HIIE consisting of a 10 min warm-up followed by 10 s sprints every 2 min for 24 min and then an 11 min cool-down performed in a fasting vs. postprandial state resulted in a different BG trajectory [65]. As seen in our study, the postprandial HIIE significantly reduced glycaemia (pre- vs. immediately post-exercise) in T1D patients, and this glycaemia-lowering effect was maintained for 24 h. Fasting HIIE, on the other hand, led to an increase in BG, where the authors speculated that such a difference could have been associated with the dawn phenomenon and a greater secretion of growth hormone in the morning, having a lipolysis-enhancing and hence glucose-sparing effect during the morning HIIE [66,67]. In our study, the participants performed HIIE in a post-prandial state, and hypoglycaemia developed at least once (the participants monitored their glycaemia at a varied frequency) in 20% of T1D patients on the day after completing HIIE, with the same percentage of occurrence the following day. Yardley [65] suggested that performing HIIE in a fasted state may be beneficial to avoid hypoglycaemia during exercise. In a case study conducted by Cockroft et al. [68], HIIE consisting of a 3 min warm up at 20 W, eight bouts of 1 min cycling at 90% of peak power interspersed with 1.25 min recovery at 20 W, followed by a 3 min cool down at 20 W, caused a drop in BG in two out of three adolescents with T1D and led to a lower average glycaemia in the 24 h post-exercise period; this finding was also observed in our study. The authors compared the glycaemic effects of HIIE vs. MICT and concluded that both HIIE and MICT had the potential to improve short-term glycaemia control in young individuals with T1D, but HIIE was more enjoyable to perform as it was more invigorating and gave the participants a greater sense of accomplishment than MICT [60,68].

It has been demonstrated that, for overall glycaemic control to be improved, a skilful balance of insulin dosing and consumption of food, especially glycaemic CHO, before, during and after exercise is required from T1D patients [69,70]. In our study, it seemed that the fear of hypoglycaemia in the post-exercise period led to a greater intake of such CHO. In our opinion, patients with T1D would benefit from knowing what types of CHO should be consumed in the post-exercise period to reduce the frequency of glycaemic disorders, both hypo- and hyperglycaemic. This would also help to improve the long-term glycaemic control and maintain HbA1C at the recommended level (HbA1C < 7.0%) to reduce the risk of diabetes-related complications [23]. It has been shown that even a 0.2% reduction in HbA1C, albeit slight, reduces cardiovascular risk by 10% [71]. In our study, the baseline HbA1C was >7.0% in the majority of T1D participants (78%; seven out of nine) although it was 8.9% in one participant, which indicated poor glycaemic control [23]. There is evidence to show that low glycaemic index (GI) and glycaemic load (GL) diets improve glycaemic control in T1D and T2D, with more studies being conducted on the latter [72]. Jenkins et al. [73] demonstrated that a 3-month low-GI diet (with a high content of legumes or fibre) improved glycaemic control in adult T2D patients. Similarly, a long-term dietary treatment (20% protein, 30% fat and 50% CHO) with increased amounts of fibre-rich (50 g/day) and low-GI natural foods improved glycaemia control and decreased the number of hypoglycaemic events in T1D patients [74]. In our study, participants with T1D consumed similar total (glycaemic and non-glycaemic) CHO (54% of total energy intake); however, we did not analyse their fibre intake, which makes this comparison unreliable. The total CHO of about 54% of daily energy intake was within the European Association for the Study of Diabetes’ dietary recommendations (45–60%) [75]. However, two T1D individuals consumed larger amounts of CHO (>70% of total energy intake), and this seemed to have influenced their HbA1c, which was significantly above the recommended level (HbA1c < 7.0%) in one of these participants (HbA1c = 8.9%) [23]. This finding shows that an excessive intake of CHO has a significant impact on glycaemic control; however, insufficient glucose monitoring and insulin administration, as well as physical activity level, could have also contributed to this finding. 

It is worth noting that an excessive glycaemic CHO intake can be counteracted by exercising. It has been demonstrated that, within the physiological range of serum BG, the relationship between plasma glucose concentration and glucose uptake in muscle during exercise is almost linear [76]. As patients with T1D often have abnormally elevated BG, it is not clear whether this relationship remains linear, and it is expected that skeletal muscle glucose uptake could be limited by, for example, reduced perfusion resulting from hyperglycaemia-induced ED of the muscle vasculature [7,8,9]. Additionally, studies have shown a positive correlation between exercise intensity and rate of skeletal muscle glucose uptake, indicating that HIIE will be more effective in inducing skeletal muscle glucose uptake than MICT, which can be attributed to a greater GLUT-4 translocation, blood flow and metabolic stress [63,64]. 

Overall, the T1D participants consumed less CHO in comparison with the control group; however, the difference was insignificant. The diet of the control group also consisted of a larger amount of glycaemic CHO, such as potatoes and bread, when compared with T1D patients. Post-prandial glycaemia following a carbohydrate-rich meal, and the resultant insulinaemia, have been implicated in the aetiology of cardiovascular disease and T2D; hence, it seems reasonable to assume that lower-to-moderate intake of glycaemic CHO may protect from the development of such diseases in healthy people [77,78]. The ideal amount of CHO in the diet of patients with T1D is still unclear [79]. Studies in the U.S. have shown that most individuals with T1D and T2D report consuming moderate amounts of carbohydrate (~45% of total energy intake) [79,80], which is lower than in the present study. 

Fat is a macronutrient that can influence glycaemia, as co-ingestion of fat with CHO slows down gastric emptying and, in turn, the release of glucose into the blood which ultimately reduces BG [77]. In our study, both groups consumed low amounts of fat (both saturated and unsaturated), i.e., 19.4% of total energy intake. Similarly, data on the ideal total dietary fat content for people with T1D seems to be inconclusive (30–35%) of total energy intake for general population [79], but a Mediterranean-style diet that is rich in monounsaturated and polyunsaturated fats has been shown to improve glucose metabolism and be more beneficial than a low-fat high-carbohydrate diet [81,82]. 

Protein intake also affects glycaemia as, similarly to fat, its ingestion slows down gastric emptying, and hence glucose release into the bloodstream [83]. With research being inconclusive as to the ideal amount of dietary protein to optimise glycaemic control and/or cardiovascular disease risk in DM, protein intake goals should follow that of the general population (10–15% of total energy intake) [79,84], or thy may need to be individualised in certain cases [85]. Those with diabetic kidney disease should aim to consume no more than 0.8 g/kg body mass/day [85]. In our study, the T1D group consumed more protein than this (116.2 ± 58.4 g/day; 1.6 ± 0.8 g/kg/day; ~20% of total energy intake), as did the control group, yet participants did not have albuminuria or reduced estimated glomerular filtration rate. It is worth noting that certain amount of protein consumed is converted into BG in the process of gluconeogenesis, nevertheless its effect on BG seems to be relatively small [83]. 

The fact that there was low fat and high protein intake in both groups, with excessive intake of glycaemic CHO in some T1D individuals and most of the control group participants, deserves some attention. Dietary guidelines for T1D patients, including what to consume before and after exercise, do not seem to be fully established yet, and this requires exploration. We propose that, in addition to regular health checks related to DM, the diets of T1D patients, as well as the levels of physical activity, should be screened on a regular basis to improve glycaemic control and prevent the development of chronic complications. 

We propose that, in the post-exercise period and depending on BG levels, T1D patients could consume the following types of CHO: (1) after completion of a bout of exercise, foods that contain both glycaemic and non-glycaemic CHO, such as an apple, banana, a handful of berries, or a portion of oats; (2) 2–3 h after exercise, a meal balanced in terms of CHO, protein and fat content, where glycaemic CHO (potatoes, rice, pasta) make up about a quarter, not more. If, however, after exercise, the patient is experiencing an episode of hypoglycaemia (BG of <70 mg/dL), then easily absorbed glycaemic CHO in the form of fruit juice should be consumed [23], and if the patient is experiencing hyperglycaemia (fasting BG of >125 mg/dL or 2 h postprandial BG of >180 mg/dL), then glycaemic CHO should not be consumed for a certain period of time, depending on the BG trajectory. Generally, a glycaemic CHO intake should be carefully monitored to avoid excessive consumption, and it should be adjusted according to physical activity and exercise (patients should reduce an overall intake of potatoes, rice, bread, especially on the days of less physical activity).

We also propose that HIIE should be implemented into a daily routine of T1D patients. T1D patients often face different barriers to exercise, including difficulty with cost and travel time to gyms, limited access to stationary bikes and treadmills, and potentially not enjoying exercising in front of others [86]; hence, HIIE could be performed at home, during leisure time, and also on the way to work or during a break at work. The intensity (submaximal–maximal–supramaximal), duration and frequency of HIIE should increase gradually, including the length of exercise element vs. length of rest. Examples of implementing HIIE into a daily routine would include running instead of walking up the stairs, performing jumping jacks, hops, jogging on the spot (30 s) [86] or jumping on a skipping rope, or interspersing continuous cycling with a short (30 s–1 min) period of high-intensity cycling. 

As T1D is associated with impaired adaptive response to hypoxia, a common feature of T1D [17], and with chronic inflammation leading to the development of micro- and macrovascular complications [23], we also measured the effect of HIIE on the serum levels of selected pro-inflammatory cytokines, TNF-α, VEGF and of the main regulator of response to hypoxia, HIF-1α.

T1D had a significant effect on HIF-1α, as its resting serum levels were 20-fold higher in the T1D group compared to healthy participants. This finding confirms a stimulatory effect of hyperglycaemia-induced hypoxia on the expression of HIF-1α [15,16], as, under normoxic conditions, HIF-1α has an extremely short half-life of less than five minutes, as it is continuously synthesized and degraded [87]. The HIIE led to a 34.4% and 39.1% decline in the level of HIF-1α immediately and at 24 h after completion of HIIE, respectively. Conversely, in the control group, HIF-1α increased over 2-fold in response to HIIT, which confirms that high-intensity exercise induces hypoxia and enhances the stability, and thus the serum level, of HIF-1α [88]. The decreasing trend of HIF-1α in T1D patients may be associated with the BG decline in response to HIIE and confirms that better glycaemic control is crucial and may diminish cellular hypoxia and reduce inflammation, since HIF-1α also regulates the expression of the genes coding for pro-inflammatory cytokines: TNF-α and VEGF [89]. Li et al. [90] also observed elevated serum levels of HIF-1α in patients with T2D compared to healthy individuals; however, the mean HIF-1α concentration was substantially lower than that observed in our study (0.2 ± 0.1 ng/mL vs. 657.0 ± 210.4 ng/mL, respectively). Additionally, the T2D patients with coronary artery calcification were found to have significantly higher HIF-1α levels compared to those without, and HIF-1α correlated positively with HbA1c and other factors of inflammation (CRP, IL-6) [90], which makes HIF-1α a good candidate for a marker of inflammation and glycaemic control in DM. Rusdiana et al. [91] also demonstrated lower levels of HIF-1α in patients with T2D compared to what was observed in the current study (1.7 ± 0.6 ng/mL vs. 657.0 ± 210.4 ng/mL, respectively). The difference between HIF-1α serum levels in T1D vs. T2D could be explained by a more severe and more frequent hyperglycaemic episodes in T1D patients, and consequently, greater hyperglycaemic-induced expression of HIF-1α [15,16]. Studies demonstrating serum concentration of HIF-1α in humans with T1D seem to be lacking, but a few in vitro studies have shown augmented expression and stability of HIF-1α in cultured human retinal pigment epithelium [92]. Nevertheless, the majority of in vitro studies have concluded that hyperglycaemia is responsible for reduced HIF-1α stability and compromised transcriptional activation function via impaired interaction with the transcriptional coactivator p300 [93]. Thangarajah et al. [94], on the other hand, demonstrated that only HIF-1α activity, not stability, is impaired in the high-glucose environment (hyperglycemic culture). 

One of HIF-1α target genes is VEGF, which acts as a pro-inflammatory and pro-angiogenic cytokine, which is essential for postnatal neovascularisation [94,95]. In our study, the pre-exercise levels of VEGF tended to be lower in the T1D compared to the control group, which suggests impaired transcriptional activity of HIF-1α as demonstrated by Thangarajah et al. [94]. Interestingly, VEGF tended to increase in response to HIIE, and this observation was accompanied by decreasing levels of HIF-1α. It has been previously demonstrated that, not only HIF-1α, but also vascular sheer stress during exercise, can stimulate the expression of VEGF [96]. Moreover, Shoag and Arany [97] showed that the transcriptional coactivator PGC-1alpha (peroxisome-proliferator-activated receptor-gamma coactivator-1alpha), a major regulator of mitochondrial function in response to exercise or other situations characterised by a lack of oxygen and nutrients, stimulates VEGF expression via an HIF-1α-independent pathway in cultured muscle cells and skeletal muscle in vivo. In the present study, we did not measure the level of PGC-1α, and therefore we can only speculate that its expression increased in response to HIIE and contributed to the elevated VEGF in the T1D group. In the control group, on the other hand, VEGF tended to decrease in response to HIIE. VEGF expression has been found to be dysregulated in various tissues of T1D patients. The angiogenic paradox, where angiogenesis is either insufficient in myocardium, nerves, skeletal muscle and skin, or excessive in the retina, is a phenomenon that can occur in the same patient with T1D [98,99,100]. Since inadequate collateral blood vessel formation in response to hypoxia and reduced wound healing increases cardiovascular morbidity and mortality, and the risk of amputations, respectively, the observed tendency for VEGF to increase in response to HIIE, as noted in our study, with its level staying elevated at the 24 h, seems to be a beneficial observation in patients with T1D, in whom angiogenesis may be insufficient. On the other hand, VEGF is also a pro-inflammatory cytokine, and its elevated levels indicate inflammation. However, acute inflammation following a bout of exercise leads to regeneration of the damaged myocytes as an adaptation to exercise [101]. 

VEGF expression has been shown to be also stimulated by TNF-α [102]. In the T1D group, TNF-α tended to increase, and in the control group it tended to decrease, in response to HIIE. In another study in healthy and T2D individuals, serum levels of TNF-α did not change in response to exercise of a longer duration (25 min) and lower intensity (60% VO_2_max) compared to our exercise intervention. There was also no difference between the resting and post-exercise levels of the cytokine between the studied groups that were age-, gender-, VO_2_peak-, weight- and body-mass-index-matched [103]. In our study, the T1D patients were found to have 2-fold lower pre-exercise levels of TNF-α compared to the control group, but the difference was not significant. TNF-α is a biomarker of systemic inflammation [3,4,5]; therefore, the fact that its level was lower in the T1D group could indicate no chronic inflammation. Nevertheless, this observation is based on the mean value and 22.2% of T1D participants (*n* = 2) were found with resting TNF-α of >50.0 pg/mL, which indicated a rather high level of inflammation when compared with the normal range of the cytokine observed in individuals without inflammatory diseases [104,105]. TNF-α is amongst the main pro-inflammatory cytokines implicated in the inflammation of the pancreatic beta cells [106]; hence, its elevated level is to be expected in T1D patients. Additionally, we cannot rule out the effects that the previous exercise sessions of the project (stage 1 and stage 2) may have had on the TNF-α results (and as a matter of fact, also on VEGF and HIF-1α); although the sessions were seven days apart, this is unlikely. The decrease in TNF-α response to HIIE observed in the control group could be explained by a higher level of aerobic fitness (based on VO_2_peak) and greater adaptation to high-intensity exercise of the healthy individuals compared to the T1D group, and hence a diminished inflammatory response to a single exercise session [101]. 

## 5. Conclusions

In conclusion, our study demonstrates that a bout of high-intensity interval exercise is effective in both reducing glycaemia of patients with T1D to a safe level and in improving short-term glycaemic control. The effectiveness, short-duration and potential more enjoyment from performing interval exercise have practical implications, and implementing this type of exercise into daily routine may be more achievable for T1D patients rather than implementing time-consuming MICT. On the other hand, these exercise modalities could be combined into one exercise session. 

An excessive intake of digestible carbohydrates in the post-exercise period (24 h after the exercise) diminishes the glycaemia-lowering effect of the exercise, but it appears to be driven by the fear of hypoglycaemia. Diabetes was associated with abnormally elevated serum levels of the main mediator of adaptive response to hypoxia (HIF-1α), which develops in a hyperglycaemic environment that impairs its transcriptional activity. The high-intensity interval exercise led to an increase in the main stimulator of angiogenesis (VEGF), which could indicate an improved adaptive response in hypoxic tissues in patients with T1D, although the increase was insignificant. This study also highlights the need for the screening of both diet and physical activity level in T1D patients, and education to explain the type and amount carbohydrates that should be consumed post-exercise to minimise the frequency of acute glycaemic disorders that over time lead to the development of diabetes-related complications. 

## 6. Limitations

Due to challenges in recruiting patients with T1D who were willing to participate in our study, the sample size is small. The intensive insulin therapy and different types of insulin and methods of its administration could have affected the glycaemic response to the exercise intervention in the T1D group. Moreover, we did not measure the concentrations of the counterregulatory hormones (noradrenaline, adrenaline, cortisol, growth hormone), which further impedes the interpretation of the HIIE effects. Finally, the serum concentrations of HIF-1α, TNF-α and VEGF were assessed immediately after and at the 24 h post-exercise, which limits the ability to draw conclusions about the long-term therapeutic effects.

## Figures and Tables

**Table 1:** Somatic and physiological characteristics, and macronutrients intake of the studied groups.

	T1D Group *n* = 9 (Female *n* = 2)				Control Group *n* = 9 (Female *n* = 3)			
	Mean	±SD	Min	Max	Mean	±SD	Min	Max
Age	30.2	10.9	18.0	43.0	22.4	1.4	21.0	26.0
Body mass (kg)	74.8	14.3	51.1	99.1	70.1	9.2	58.3	91.5
Body height (m)	1.8	0.1	1.6	1.9	1.7	0.1	1.6	1.8
BMI (kg/m^2^)	23.8	3.6	18.1	29.9	23.5	2.5	19.9	27.9
%BF	17.5	8.3	6.5	33.5	18.7	6.3	10.1	26.4
FFM (kg)	61.6	12.6	41.6	75.3	56.9	8.3	43.4	70.0
VO_2_peak (mL/kg/min)	39.0	8.2	31.0	48.0	47.8	11.1	30.0	69.0
T1D duration (years)	12.3	9.1	4.0	27.0	n.a.	n.a.	n.a.	n.a.
HbA1C (%)	7.3	0.8	6.1	8.8	n.m.	n.m.	n.m.	n.m.
Energy intake (kcal/day)	2153.7	739.1	1076	3243	2778.6	769.1	2174.7	4408.9
CHO intake (g/day)	241.3	98.2	113.7	365.5	353.2	146.9	250.4	663.6
CHO %energy intake	54.0	10.3	41.2	70.8	60.4	6.9	48.5	70.0
Protein intake (g/day)	116.2	58.4	51.2	224.7	111.4	27.6	82.1	159.0
Protein intake (g/kg/day)	1.6	0.8	0.7	2.8	1.6	0.4	1.2	2.2
Protein %energy intake	26.6	9.8	13.5	42.3	20.2	5.6	13.9	30.8
Fat intake (g/day)	86.2	45.0	25.8	152.7	109.2	22.6	76.7	152.1
Fat %energy intake	19.4	7.1	10.3	29.5	19.4	2.2	16.1	22.9

BMI—body mass index; %BF—percentage of body fat; FFM—fat free mass; T1D—type 1 diabetes; HbA1C—glycated haemoglobin; CHO—carbohydrates; n.a.—not applicable; n.m.—not measured.

**Table 2:** Fasting glycaemia, insulin administration and digestible carbohydrates intake on the day of (day 1) and the day after (day 2) completion of HIIE in T1D group.

Variables	T1D Group	
	Mean	SD
Fasting glycaemia day 1 (mg/dL)	164.5	78.5
Fasting glycaemia day 2 (mg/dL)	139.0	9.9
Insulin day 1 (units/day)	35.3	10.9
Insulin day 2 (units/day)	34.5	13.5
Digestible CHO day 1	157.0	104.2
Digestible CHO day 2	190.5	57.7

**Table 3:** Serum levels of the transcription factor HIF-1α and pro-inflammatory cytokines (TNF-α and VEGF) in response to HIIE in the T1D and control group.

Variables	T1D Group		Control Group		T1D Group		Control Group	
	Mean	±SD	Mean	±SD	%Change: Rest vs. Max	%Change: Rest vs. 24-h	%Change: Rest vs. Max	%Change: Rest vs. 24-h
**HIF-1α rest (ng/mL)**	657.0 ***	**210.4**	**28.3**	11.5	34.4% ↓	39.1% ↓	278.9% ↑	242.7% ↑
HIF-1α max (ng/mL)	430.0	163.3	107.0	56.9				
HIF-1α 24 h (ng/mL)	399.5	297.7	96.8	76.9				
TNF-α rest (pg/mL)	17.6	19.4	37.8	13.0	111.0% ↑	123.1% ↑	26.3% ↓	44.3% ↓
TNF-α max (pg/mL)	37.2	9.3	27.7	10.3				
TNF-α 24 h (pg/mL)	39.3	16.5	20.9	9.5				
VEGF rest (pg/mL)	7.3	4.6	16.2	4.6	310.3% ↑	103.4% ↑	62.9%↓	61.4% ↓
VEGF max (pg/mL)	30.0 *	25.3	6.1	3.4				
VEGF 24 h (pg/mL)	14.9	3.1	6.3	3.2				

Max—immediately after HIIE; 24-h—24 h after HIIE; ↑—increase; ↓—decrease; * *p* < 0.05, *** *p* < 0.001—significant difference between the T1D group and control group.

## Data Availability

The datasets used are available from the corresponding author upon request.
